# HIV Plays (and Wins) a Game of T Cell Brinkmanship

**DOI:** 10.1371/journal.pbio.1001521

**Published:** 2013-04-02

**Authors:** Roland G. Roberts

**Affiliations:** Public Library of Science, Cambridge, United Kingdom

Viruses are locked into an adaptive arms race with the host immune system: the immune system adapts to recognize the virus, the virus adapts to evade the immune system, the cycle repeats. But in the case of HIV, it seems, sometimes it's good to be recognized.

**Figure:**
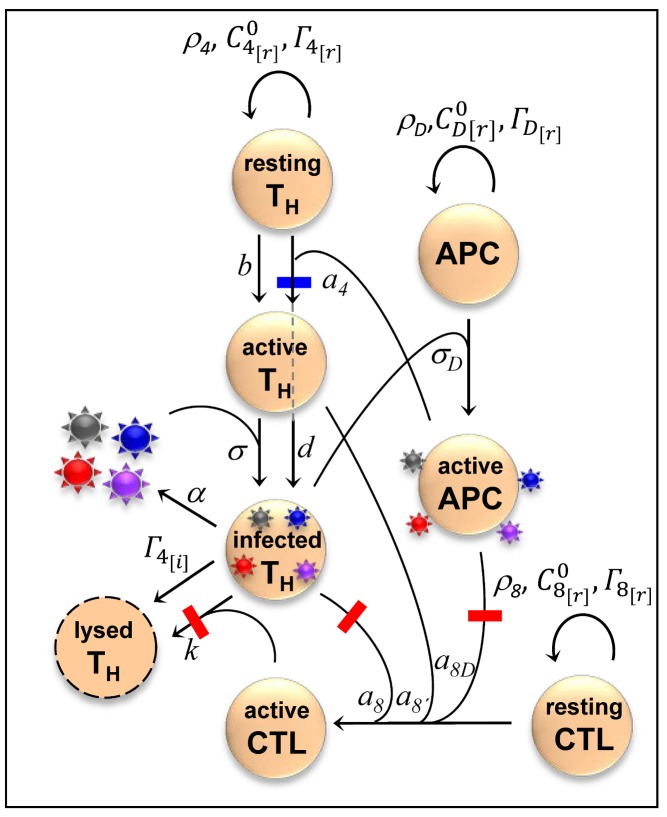
A mathematical model captures many of the complexities of HIV's relationship with the human immune system, revealing that sometimes the benefits of detection by T_H_ cells can outweigh the costs of detection by cytotoxic T cells (CTLs).

The cellular branch of the immune system comprises T cells that recognize specific short peptides (epitopes) clipped from viral proteins and displayed on the surface of infected cells. Like antibodies, the receptors borne by T cells are “adaptive”, that is, they can vary and be selected for if they detect something useful (like viruses). As ever with the immune system, things are of course somewhat more complicated—there are several major types of T cells that do different jobs. The ones that concern us here are the T helper cells (T_H_ cells), which need to be activated by epitopes displayed on the surface of “professional antigen-presenting cells”, and can then collaborate with another type of T cell—the cytotoxic T cells (CTLs)—to destroy infected cells.

This means that there's a strong incentive for the corresponding epitopes of the viral proteins to mutate, allowing the virus to escape T cell surveillance and survive for longer in the host body. As a result, most viral genomes tend to vary substantially in the regions that encode T cell epitopes. But HIV stands out as an exception to this tendency—their T cell epitopes seem to vary *less* than the rest of the genome. Why?

Several theories have been advanced to explain this counterintuitive result, but a new study by Rafael Sanjuán, José Alcamí, and colleagues in *PLOS Biology* provides evidence for a tantalizing and potentially important new explanation. It revolves around another peculiarity of HIV—the fact that it infects the very cells that are responsible for the cellular immune response—the T cells themselves.

The authors carefully scrutinized a wide range of HIV genome sequences to look for patterns of variation. For comparison, they also checked sequences of the more conventional hepatitis C virus (HCV). HCV doesn't infect T cells, and its T cell epitopes vary in an attempt to fly under the T cell radar. HIV does infect T cells, and its T cell epitopes are puzzlingly unchanging. After ruling out a number of alternative explanations, the authors set up a mathematical model to test a hunch.

The hunch is that HIV may not always want to go unnoticed. It's known that HIV replicates more efficiently in T_H_ cells that have been activated, and this presents the virus with a bit of a dilemma—it certainly doesn't pay to be recognized by CTLs, the hired assassins of the immune system. But there's also a possible benefit in triggering the activation of the T_H_ cells that they've infected. Where does the balance lie? Is it conceivable that there are some conditions where it's better for HIV epitopes to be recognized than to be ignored?

In answer, the authors model the complex network of interactions between T_H_ cells, CTLs, antigen-presenting cells and viruses (see image). They set up two different versions of the model—one with a virus that infects non-immune cells (like HCV infecting hepatocytes), and one with a virus that infects T cells (like HIV). The results are quite striking—in the case of HCV, evasion always pays off. But in the case of HIV, the dependence on T_H_ activation seems to sometimes favor viral epitopes that are strongly recognized by cellular immune system.

Using their combination of sequence data analysis and mathematical modeling, the authors propose that recognition of HIV epitopes by T_H_ cells can be beneficial for the long-term survival of the virus within the body. Conditions identified in the model suggest that this recognition exerts its influence during the process of “trans-infection”, a feature of the chronic stage of HIV-related disease whereby T_H_ cells become infected during their activation in the lymph nodes. Another key variable is how many T_H_ cells are activated in the body by pathogens other than HIV. If this “background activation” is strong, HIV obtains no payoffs from being recognized. Studying the sequence data confirms that in the capsid genes (Gag) it is T_H_ epitopes, rather than CTL epitopes, that are constrained and that epitopes stay the same within patient viral populations rather than between patients, again implicating chronic internal transmission between cells.

The authors' hypothesis highlights the intriguing effects of opposing selective forces on HIV genome sequence, but they also point out that there is a potentially important practical implication of their findings. If their model is correct, then HIV vaccines based on T_H_ epitopes might prove counterproductive, playing into the hands of the virus. Instead, vaccines should target only CTL-specific viral epitopes.

**Sanjuán R, Nebot MR, Peris JB, Alcamí J (2013) Immune Activation Promotes Evolutionary Conservation of T-Cell Epitopes in HIV-1. doi:10.1371/journal.pbio.1001523**

